# Prey Capture Ecology of the Cubozoan *Carukia barnesi*

**DOI:** 10.1371/journal.pone.0124256

**Published:** 2015-05-13

**Authors:** Robert Courtney, Nik Sachlikidis, Rhondda Jones, Jamie Seymour

**Affiliations:** 1 Australian Institute of Tropical Health and Medicine, James Cook University, Cairns, Queensland, Australia; 2 NGS aquatic, Kota Kinabalu, Sabah, Malaysia; 3 College of Marine & Environmental Sciences, James Cook University, Townsville, Queensland, Australia; The Evergreen State College, UNITED STATES

## Abstract

Adult *Carukia barnesi* medusae feed predominantly on larval fish; however, their mode of prey capture seems more complex than previously described. Our findings revealed that during light conditions, this species extends its tentacles and ‘twitches’ them frequently. This highlights the lure-like nematocyst clusters in the water column, which actively attract larval fish that are consequently stung and consumed. This fishing behavior was not observed during dark conditions, presumably to reduce energy expenditure when they are not luring visually oriented prey. We found that larger medusae have longer tentacles; however, the spacing between the nematocyst clusters is not dependent on size, suggesting that the spacing of the nematocyst clusters is important for prey capture. Additionally, larger specimens twitch their tentacles more frequently than small specimens, which correlate with their recent ontogenetic prey shift from plankton to larval fish. These results indicate that adult medusae of *C*. *barnesi* are not opportunistically grazing in the water column, but instead utilize sophisticated prey capture techniques to specifically target larval fish.

## Introduction

Cnidarians utilize a diverse array of food acquisition/prey capture strategies ranging from reliance on symbiotic zooxanthellae and filter feeding, to active prey capture with nematocyst laden tentacles [1–4]. Those that use nematocysts may implement simple prey capture strategies which rely on size and tentacle structure to opportunistically graze within the water column [5], while others use propulsion and induced swimming kinematics to increase potential prey and food particle contact with trailing tentacles [6]. Others, such as Cubozoans, are highly mobile and posses complicated visual structures, which have been hypothesized to play a role in prey capture [7–10].

Perhaps the most extreme prey capture strategy recorded so far is seen in Siphonophores, which use modified tentacles as ‘lures’ in a form of aggressive mimicry [11]. They actively attract and lure specific prey types, either through resembling schooling conspecifics, or by mimicking the prey items of the targeted species [3,11–13]. Many Siphonophore lures not only mimic the appearance of other species but also their movements. Specifically, these lures are motile and are often moved using a ‘jigging’ or ‘twitching’ motion, which resembles the movements of specific prey types [3,11].

In many Cnidarians, the diurnal light-dark cycle often mediates a condition-specific behavioral response. For example, numerous Anthozoan and Siphonophore species extend their feeding tentacles only during the day while others only at night [14–16]. Similarly, many species of Hydromedusae and Scyphomedusae use light to facilitate vertical migrations in the water column in order to locate food, while others laterally migrate with the sun to increase solar exposure for symbiotic zooxanthellae [17,18]. Cubozoans also undergo a diurnal behavioral shift [19,20], and vision seems to be one of the factors involved in this diurnal differentiation in behavior [21,22].

Interestingly, a variety of visual systems are utilised by Cnidarians. These range from simple eye spots and pigment cup ocelli to advanced pigment cups with lenses [23–28], with the most advanced visual sensory structures belonging to the medusa stage of the Cubozoans [24,29]. However, there has been contention as to the usefulness of these complex eyes, due to the apparent lack of either neural branches to process the information [30], or a nervous system able to interpret visual images [31].

Cubomedusae do however exhibit many light mediated behaviors [32–34]. These include targeting light shafts for feeding [7], obstacle avoidance [35], actively swimming away from dark objects [36], or decreased activity at night [20]. However, the extent that vision is used in prey capture by Cubozoans is unknown. One highly venomous Cubozoan that possesses sophisticated visual organs is *Carukia barnesi* Southcott, 1967 [24,33,37,38]. The general ecology and biology of *C*. *barnesi* is not well understood. The medusa stage of this species is seasonally present coastally along north-eastern Australia typically from November to May each year. This species is considered oceanic and is found around coral reefs and islands, and under certain conditions, on beaches [39–42]. Juvenile *C*. *barnesi* feed predominantly on crustaceans and during maturation undergo an ontogenetic venom change, correlated with a prey shift, from planktonic invertebrates to larval fish [38].

*Carukia barnesi* have four sets of six ‘eyes’, which is typical among Cubozoans, consisting of a pair of simple light sensitive pigment cups, a pair of light sensitive pigment slits, and a pair of complex eyes that each has a cornea, a lens, and a retina [24,29,36,37]. However, the acuity and use of these eyes is unknown. The complete lifecycle and feeding ecology of this species is poorly understood and has not been described to date. This study describes part of the feeding ecology of the Cubozoan *C*. *barnesi* and aims to understand the mechanisms employed by this species to capture its prey.

## Method

### Species Description

*Carukia barnesi* is a small (approximately 20 mm bell-width), oceanic, planktonic Carybdeid that inflicts a potentially fatal sting that causes Irukandji Syndrome [39–43]. This species has four tentacles in total and each extends from a pedalia attached to each corner of the bell. These tentacles are up to 750 mm long and have an alternating pattern of large and small nematocyst clusters often referred to as nematocyst-bearing rings or crescents [38,44,45]. These are referred to in this paper as large and small ‘nematocyst clusters’. The bell sizes of the specimens used in these experiments ranged from 8 to 21 mm niche bell (Nb) height (a longitudinal measure from the center of the rhopalial niche to the apex of the bell).

### Specimen Collection

No specific permissions were required for the collection locations/activities as the species involved is not endangered or protected and the collection site did not require permits. Medusae of *C*. *barnesi* were collected near Double Island, North Queensland, Australia (16°43.5′S, 145°41.0′E) during November 2013, between 1900 and 2200 h. To attract medusae, high-powered LED lights were submerged on each side of a small (five meter) research vessel. Medusae were captured as they approached the light and were transferred into individual 500ml plastic containers. The sea surface temperature varied from 27.5°C to 30°C with an average salinity of 35‰. The water depth at the capture sites varied between three to six meters. Post capture, specimens were transported to the laboratory and placed in a constant temperature controlled cabinet set at 28°C for a minimum of six hours prior to the commencement of experimental trials.

### Experimental Tank

Specimens were housed in a purpose-built plankton kreisel (a circular tank [1170 mm X 400 mm wide with an effective volume of ~ 375 liters] in which seawater rotates vertically). Seawater was maintained at 35‰ and 28°C, to mimic oceanic conditions at the specimen capture site. A photoperiod of 13 h light:11 h dark was maintained, with the light period occurring from 0600 to 1900 h to simulate the local November photoperiod. The illumination cycle was achieved by fixing lights on each side of the kreisel that provided an average light intensity of 21μmol photons/s/m^2^, and dark, achieved by turning the lights off with an electronic timer. An infra-red sensitive digital video camera was positioned approximately one meter from the face of the kreisel. Five infra-red spotlights, which remained on continuously, were positioned around the kreisel to allow for filming in darkness.

### Size Dependent Tentacle Morphology

In order to determine the relationship between medusae bell size and tentacle length, two *C*. *barnesi* were placed into the kreisel around midday and allowed to acclimate for approximately six hours prior to each experimental trial. The specimens were then filmed for 24 hours (i.e., a full 13:11 light:dark cycle) beginning with the dark cycle. This was repeated three times, with newly captured specimens, resulting in 24 hour video sequences for six *C*. *barnesi* medusae. Recorded video sequences were subsequently analyzed in 30 to 60 minute increments, where each tentacle was measured from the pedalia to its terminal end. As identifying individual tentacles on each specimen between time sequences was impossible, all tentacles at any one time were measured and the mean tentacle length of each specimen was calculated. In order to elucidate whether larger specimens, with larger bells, had longer tentacles, regression analysis was used to determine the relationship between bell size (niche bell height mm) and the maximum recorded mean tentacle length of each specimen over 24 hours. Similarly, in order to quantify the relationship between bell size and the distance between the large nematocyst clusters, the distance between six consecutive large nematocyst clusters on an individual extended tentacle were measured to the nearest millimeter for each specimen. The mean large nematocyst cluster distance for each specimen was then calculated and regressed against animal size (niche bell height mm), to determine the relationship between bell size and the distance between the large nematocyst clusters.

### The Influence of Light on Tentacle Extension

The effect of the light on tentacle extension (i.e., zero percent extension = shortest and 100% extension = longest tentacle length of each specimen) was determined at 0, 30, 60, 120, 240 and 360 minutes of exposure to both light and dark treatments. These values were arcsine square root transformed (to normalize proportional data) prior to analysis, which consisted of a two-way repeated measures ANOVA to determine if: a) light or dark; or b) the amount of time exposed to light or dark; or c) an interaction between the two, affected the percent tentacle extension. All statistical analyses were conducted using the statistics package IBM SPSS.

### The Effect of Bell Size, Light and Tentacle Extension on ‘Twitch Rate’

During the previous experiments, the extended tentacles would frequently contract in a jerking or ‘twitching’ motion, and then relax. To quantify the occurrence of these twitches, one minute sections of the video footage were analyzed in nine approximately 30-minute intervals for each specimen (i.e., 6 specimens measured 9 times each). The number of these contractions, or ‘twitches’, during each one minute sample was recorded. A ‘twitch’ was counted any time a tentacle rapidly contracted in a distinct pulse during each one minute measure, resulting in a value for ‘twitch rate per minute’ (see S1 Video). This was conducted for each specimen, and only footage from the light treatment was analyzed as twitching was not recorded in dark conditions (refer to Experimental Tank section). Linear regression was performed in order to determine if the rate of these twitches was affected by the state of a specimens tentacles (i.e., percent extended). Following this, to elucidate whether this relationship was driven by a specimen size effect (bell size), percent tentacle extension was pooled into 10 percent groups (i.e., 0–10%, 11–20%, etc.) and an ANCOVA conducted to determine if tentacle extension influenced the twitch rate, with bell size set as the covariate.

### Prey Capture

On three occasions, two *C*. *barnesi* medusae were housed in the kreisel with approximately ten larval/juvenile fish (*Acanthochromis* sp.; approximately 10–15 mm in length). The larval/juvenile fish were reared, housed and used as outlined in the ethics approval “Approval for Animal Based Research or Teaching—A2061” (granted to Dr Jamie Seymour by the James Cook University Animal Ethics Committee). Breeding pairs of *Acanthochromis* sp. were held in a 3,000 liter tank and the larval/juvenile fish feed naturally on crustaceans, such as copepods and amphipods, which occur within the 50,000 liter recirculating aquarium system. All fish used were free feeding at the beginning of this study and were only held with the *C*. *barnesi* for approximately 10 minutes per feeding event. Fish that were stung were consumed by the *C*. *barnesi* and any fish that were not consumed were returned to the larval/juvenile fish tank. During these feeding events the fish were seen to rapidly move towards the twitching tentacles and were subsequently envenomed and caught. This process was captured on video on numerous occasions and still images were extracted to investigate the prey capture method implemented by *C*. *barnesi*.

## Results

### Size Dependent Tentacle Morphology

**Fig 1:**
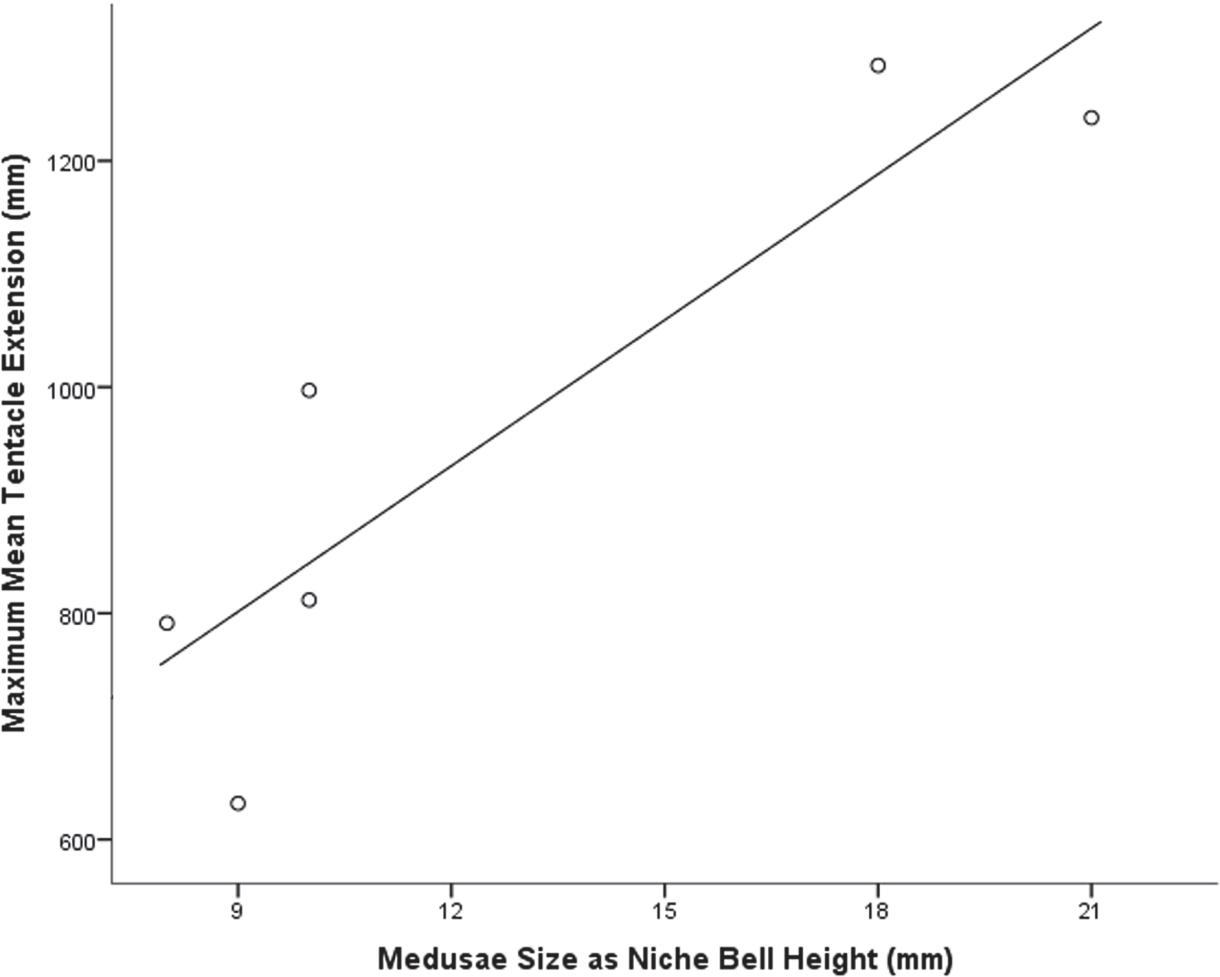
Maximum mean recorded tentacle lengths (mean length mm, n = 6) for *Carukia barnesi* over a range of medusae bell sizes (niche bell height mm).

There was a statistically significant positive relationship between bell size (niche bell height mm) and tentacle length, where larger specimens had longer tentacles than smaller specimens (*R* ^*2*^ *=* 0.796, *F*_1, 4_ = 16.642, *p* = 0.017) (see Fig 1). Conversely, the distance between the large nematocyst clusters was not correlated with bell size (*R* ^*2*^ *=* 0.003, *F*_1, 4_ = 0.13, *p* = 0.916), with a mean distance of 30 mm (± 6 mm 95%Cl) on extended tentacles.

### The Influence of Light on Tentacle Extension

**Fig 2:**
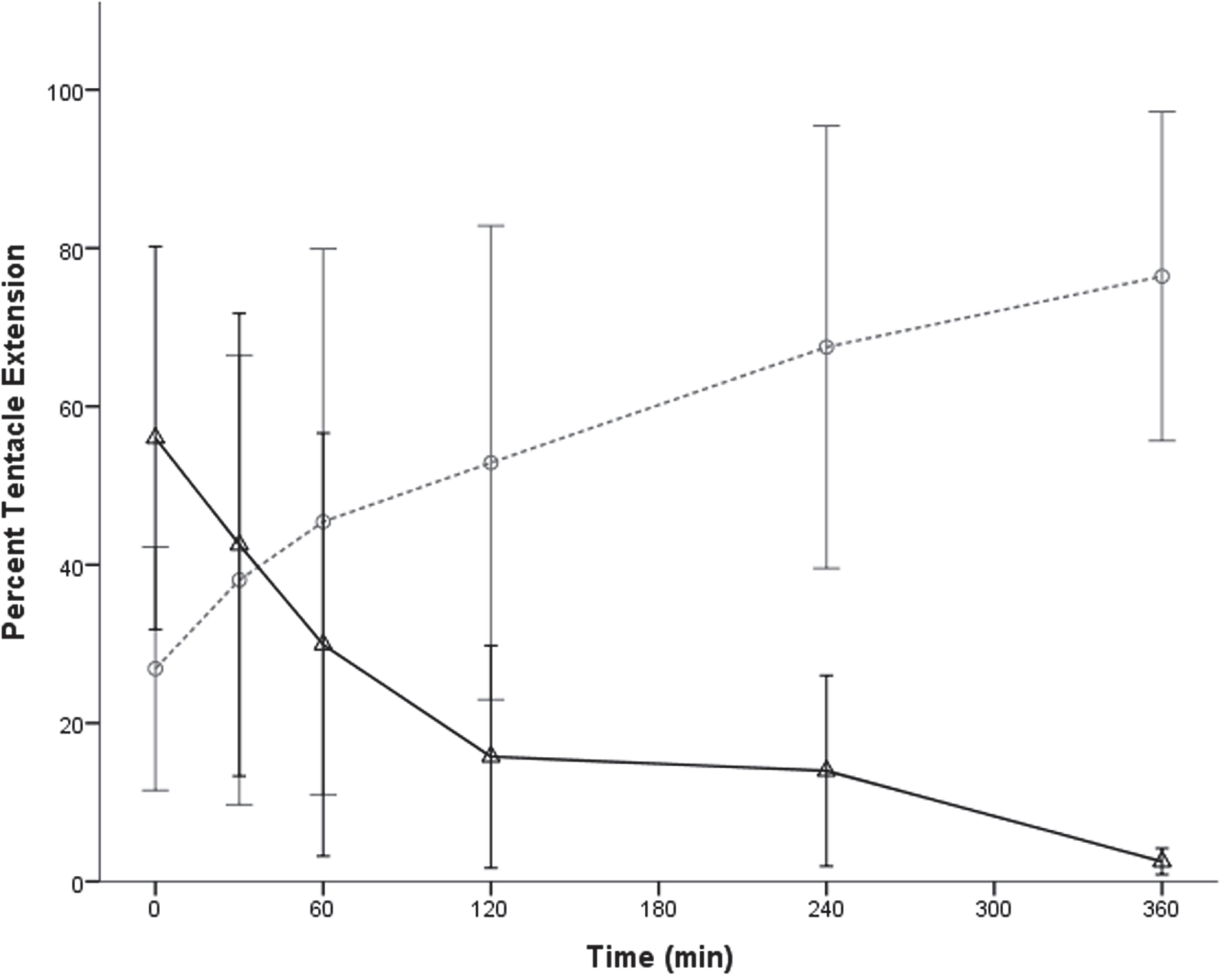
*Carukia barnesi* mean tentacle extension, as a percentage, over 360 minutes, exposed to light (○, dashed line) and dark (△, solid line) treatments. Error bars represent 95% confidence intervals (n = 6).

*Carukia barnesi* tentacles were significantly longer during the light treatment than in the dark treatment (*F*_1, 5_ = 7.112, *p* = 0.045), while time had no significant effect (*F*_5, 25_ = 0.216, *p* = 0.952). However, there was a significant interaction effect between the light/dark treatments and time (*F*_5, 25_ = 10.100, *p* < 0.001). Tentacles contracted as the dark treatment progressed reaching a contracted state of less than 20% after approximately two hours. After the lights were turned on, tentacles began to extend reaching maximum mean extension of approximately 80% after approximately six hours exposure to the light treatment (see Fig 2).

### The Influence of Bell Size, Light and Tentacle Extension on ‘Twitch Rate’

**Fig 3:**
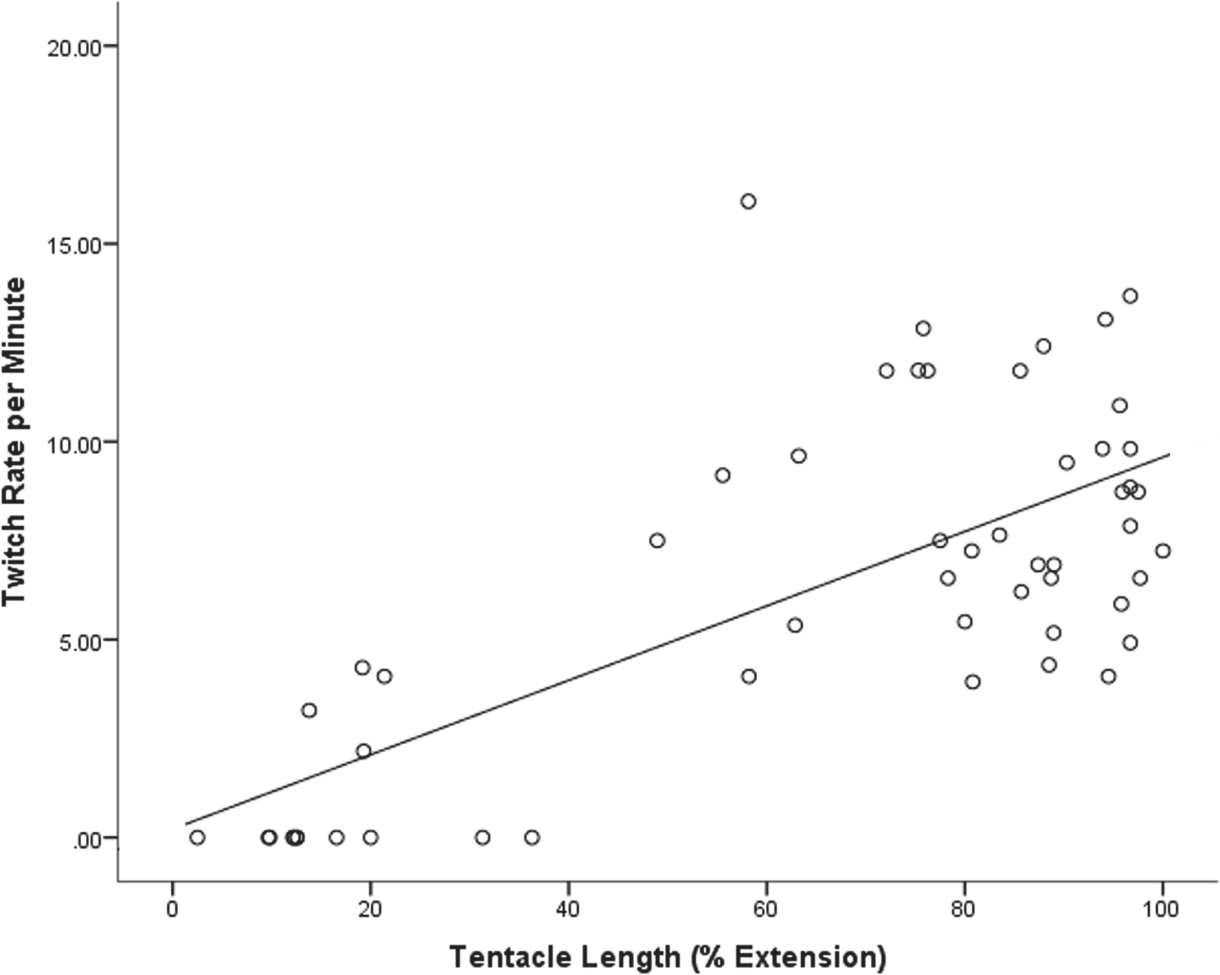
The number of tentacle twitches recorded for *Carukia barnesi*, over one minute intervals (twitch rate per minute), plotted against different tentacle extensions (percent extension). Video footage was analyzed in approximately 30 minute intervals (i.e. 6 specimens measured 9 times each).

**Fig 4:**
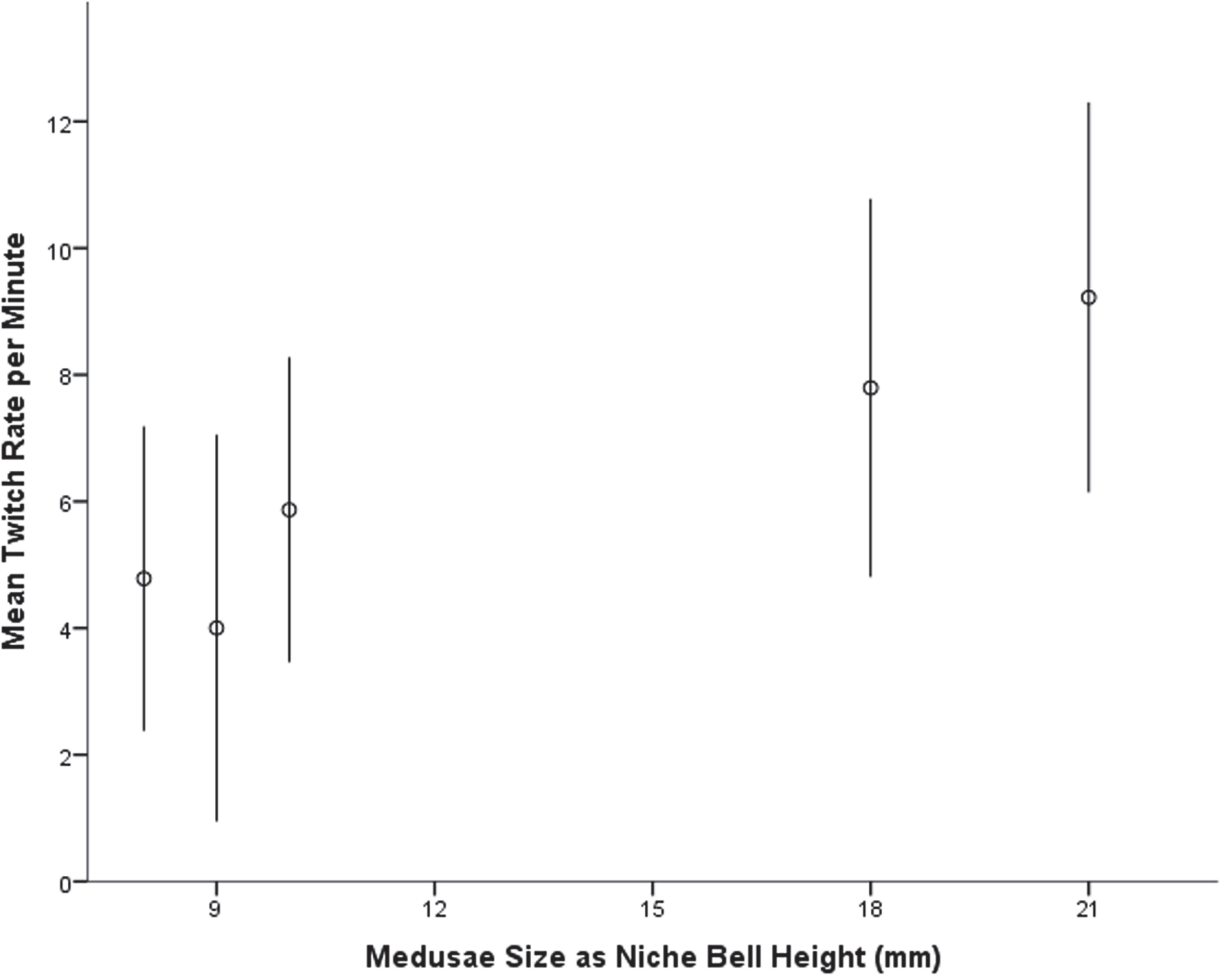
The mean number of tentacle twitches recorded for *Carukia barnesi*, over one minute intervals (twitch rate per minute) plotted against medusae bell size in millimeters. Error bars represent 95% confidence intervals (n = 6).

There was a significant positive relationship between tentacle extension and twitch rate, with elongated tentacles twitching more frequently (twitches per minute) than retracted tentacles (*R* ^*2*^ *=* 0.492, *F*_1, 53_ = 50.397, *p* < 0.001) (see Fig 3), while bell size also effected the twitch rate (*F*_4, 54_ = 2.718, *p* = 0.040), where larger animals ‘twitch’ their tentacles more frequently than small specimens (see Fig 4). The average twitch rate during the light was 6.3 twitches per minute however no twitching was recorded during the dark.

### Prey Capture

**Fig 5:**
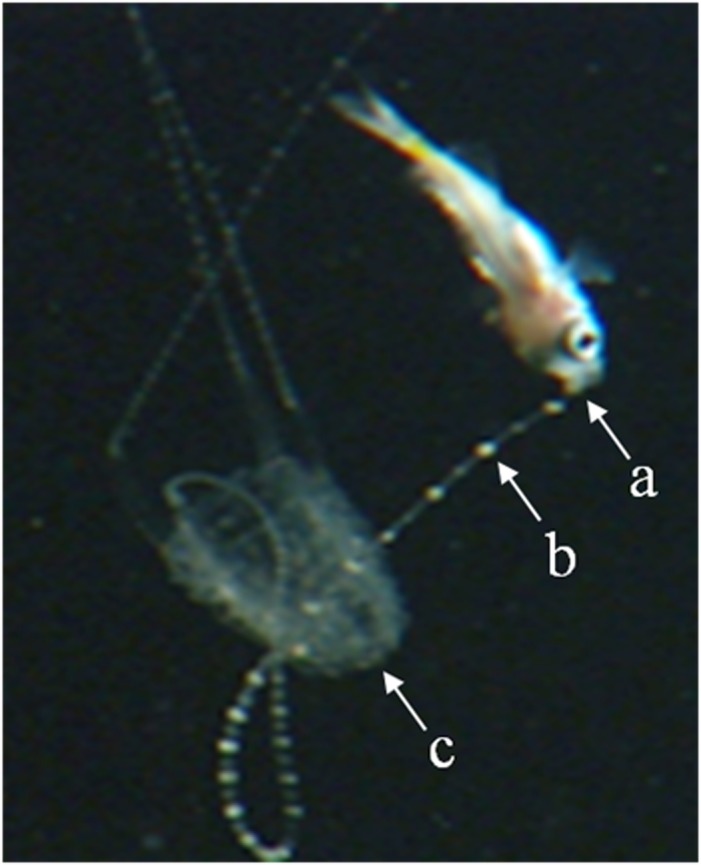
Envenomation of a larval fish (*Acanthochromis* sp.) that was captured by a twitching tentacle of an adult *Carukia barnesi*. a: envenomation site; b: nematocyst cluster; c: bell. The bell size of this specimen is approximately 15 mm in height and the fish is approximately 10 mm in length.

Larval fish (*Acanthochromis* sp.) were often attracted to the nematocyst clusters on the extended fishing tentacles of *C*. *barnesi* especially when they were being ‘twitched’. Fish would pursue these clusters and become ‘stung’ around the mouth region or head, resulting in death (see Fig 5 and S2 Video).

## Discussion

Adult *C*. *barnesi* are known to feed almost exclusively on larval fish [38]; however, their mode of prey capture seems more complex than previously described. Our findings suggest that *C*. *barnesi* are active predators that capture visually orientated prey, in this case larval fish, by using a lure-like system to simulate the size and movements of the fish’s prey (e.g., small plankton). This method of prey capture has previously been described in some Siphonophores [3,11], and considered unique compared to other Cnidarians. Many larval fish are visual hunters and because of this feed predominately during light conditions [46], and this correlated well with the luring behavior seen in *C*. *barnesi* that occurs only during daylight hours. Luring at night would be less efficient resulting in reduced prey capture and contracting their tentacles at night would decrease swimming induced drag, thus reducing energy expenditure. This suggested feeding cycle is consistent with the diurnal feeding cycle observed in another Cubozoan (i.e., *Chironex fleckeri*, Southcott, 1956), which is known to become inactive at night to conserve energy [20].

The nematocyst clusters along the extended tentacles are also motile, where *C*. *barnesi* ‘jig’ or ‘twitch’ these tentacles frequently. Fish, including larval fish, are known to be attracted to prey by movement, and may preferentially attack prey items of specific sizes [47]. In order to increase catch rates, twitching, or movement of the nematocyst clusters, would appear to serve this purpose, where movement of the nematocyst clusters would highlight these lures in the water column. Once larval fish are attracted to these nematocyst clusters they are consequentially ‘stung’ and consumed. Furthermore, larger *C*. *barnesi* were found to twitch their tentacles more frequently than smaller specimens, which may be related to their recent ontogenetic transition from planktonic to vertebrate prey [38]. Smaller medusae (under 8 mm) have a preference for plankton, and these prey items are almost certainly captured in a similar manner as used by other Cnidarian medusae, that is, by haphazardly encountering prey in the water column. As such, twitching of tentacles, which presumably increases energy consumption, would be inefficient for the capture of small plankton, which may explain the lower twitch rate observed in the smaller specimens (8–10 mm).

Not surprisingly, larger medusae were found to have longer tentacles, which would presumably increase their chance of prey capture and correlates with the change in prey preference from plankton to larval fish. However, it was surprising that the distance between the nematocyst clusters, or lures, was similar regardless of the length of their tentacles. This suggests that the distance between the nematocyst clusters are important for the visual stimulation of prey and/or to optimize prey capture (i.e., the lures are set at an optimum distance for prey capture). The function of the alternation between large and small nematocyst clusters along the tentacles is unknown; however, they may be used to target different sizes of larval fish by presenting a choice of lure/food particle sizes. Further research is required to determine the specific function of the large and small nematocyst clusters.

Cubomedusae have been shown to be more sophisticated in many areas of their ecology than most other Cnidarians. For example, they have elevated swimming speeds [48,49], greater vision capabilities [24,33,50], more sophisticated behaviors (e.g., sleeping) [19,20] and highly toxic venoms [40,43,51]. In conclusion, this research has demonstrated *that C*. *barnesi* utilize sophisticated prey capture techniques to actively lure prey. Future investigation into other species of Cubomedusae is now required to determine if they too employ sophisticated prey capture mechanisms.

## Supporting Information

S1 Video Quantification of a ‘twitch’ of the tentacles of *Carukia barnesi*. Note the two distinct twitch events during this 11 second video sequence. The first twitch event occurs after approximately one second of elapsed time. The second twitch event occurs eight seconds later.
(MP4)

S2 Video Envenomation of a larval fish (*Acanthochromis* sp.) that was captured by a twitching tentacle of an adult *Carukia barnesi*. The bell size of this specimen is approximately 15 mm in height and the fish is approximately 10 mm in length. Note the envenomation site is on the head region of the fish.
(MP4)
